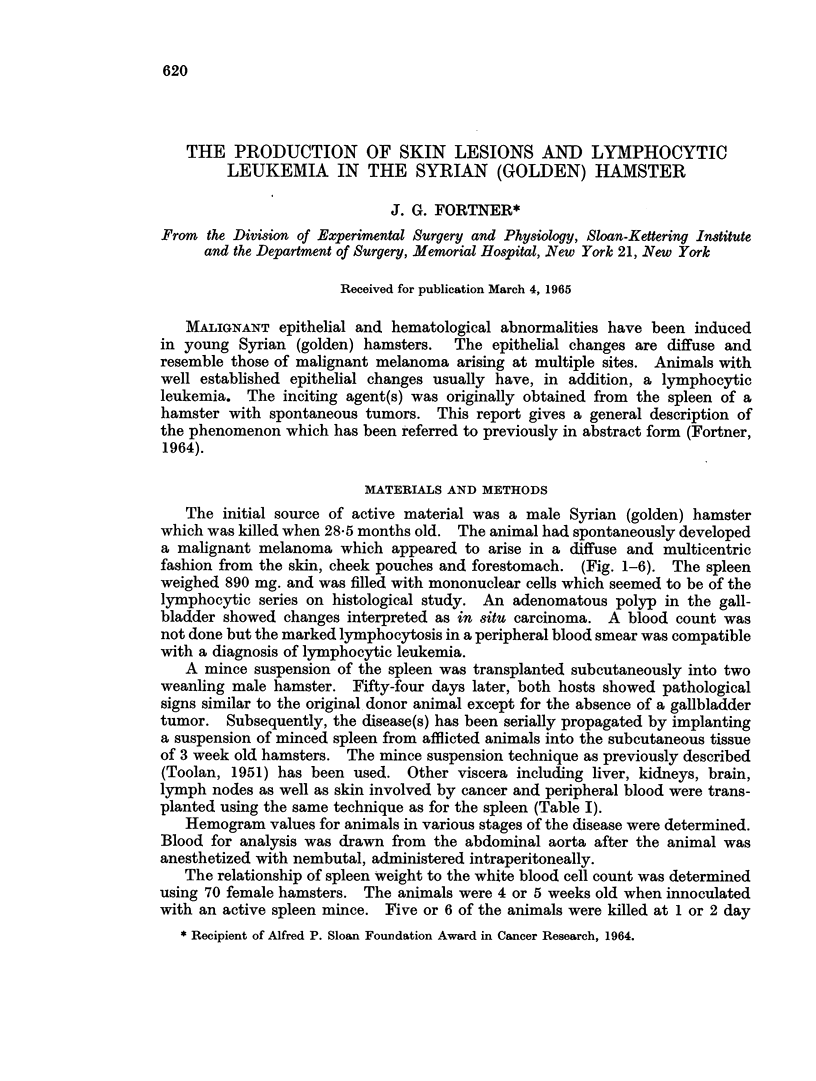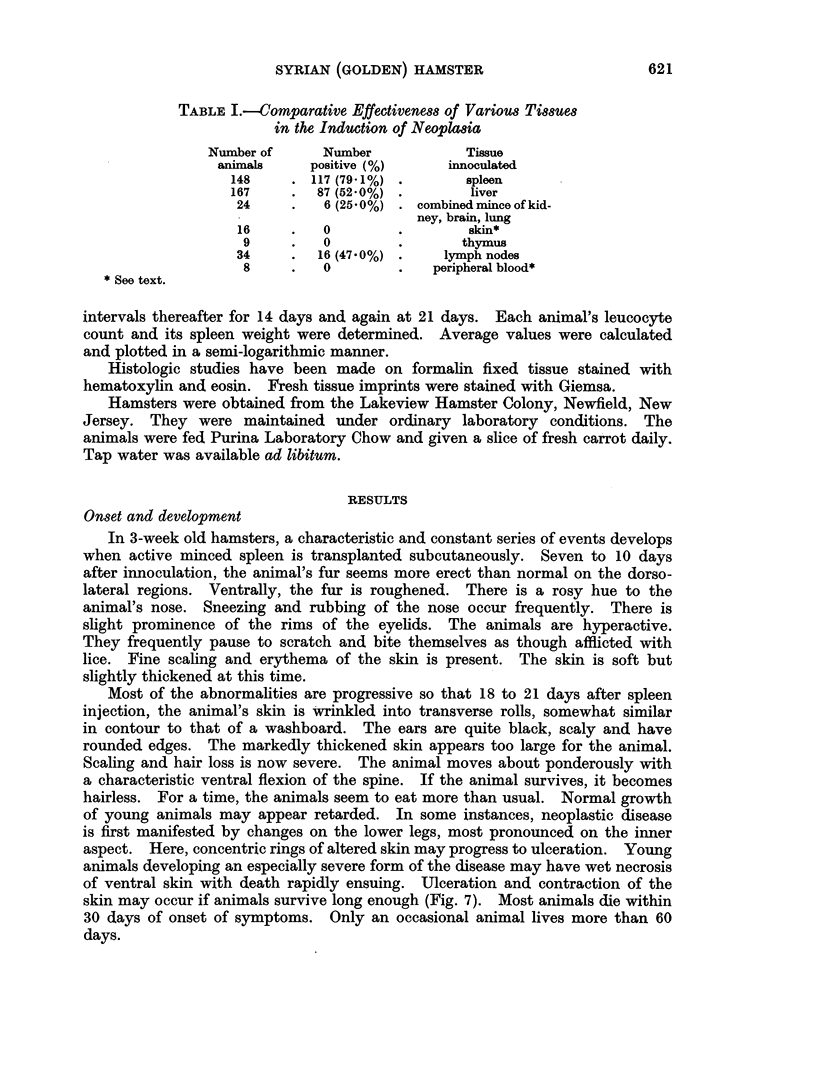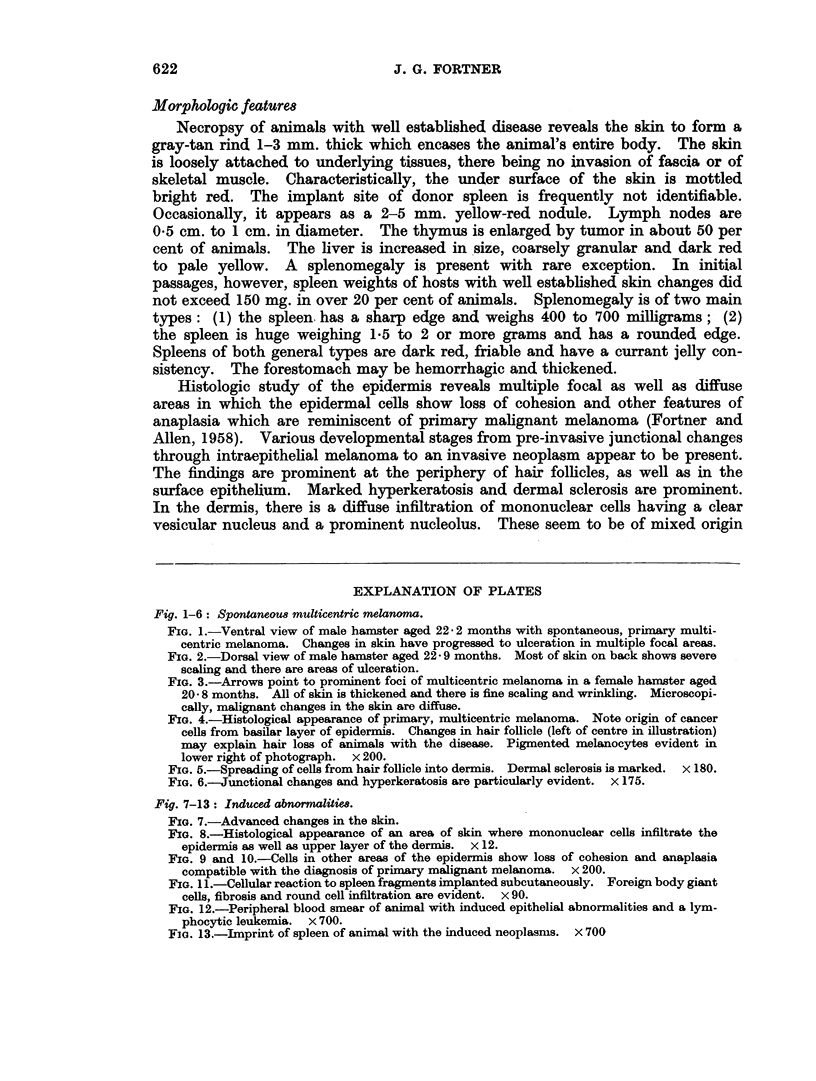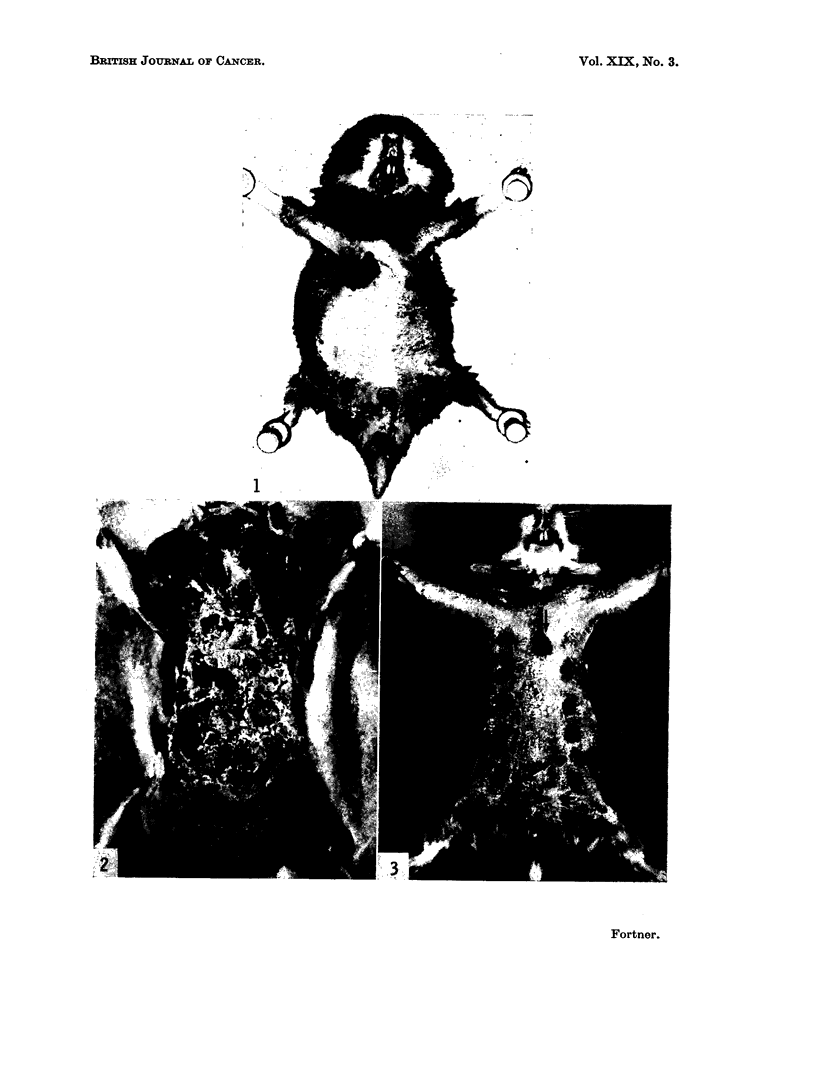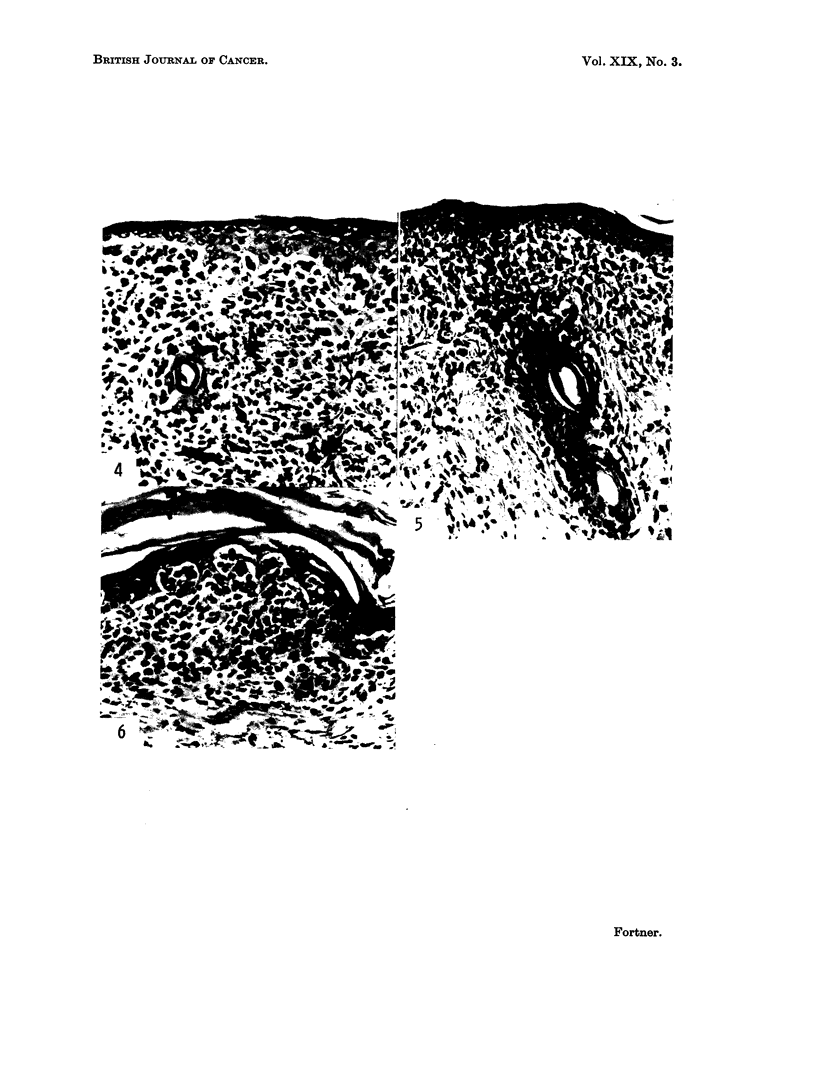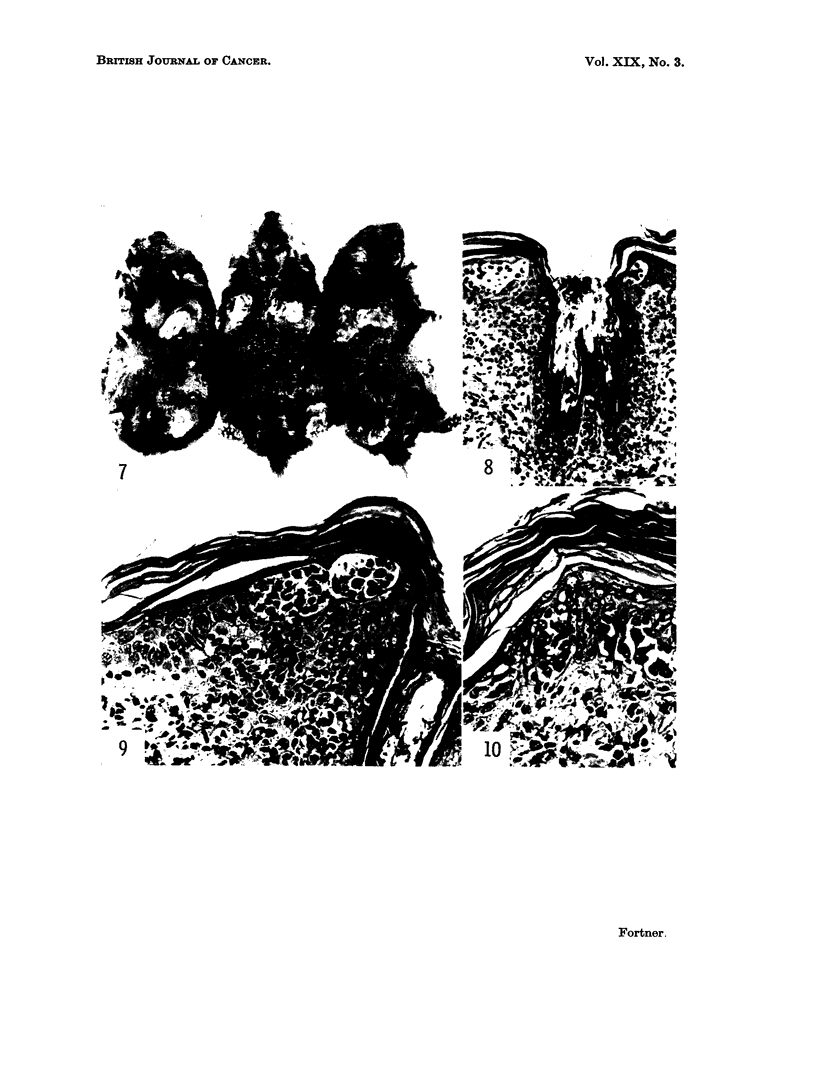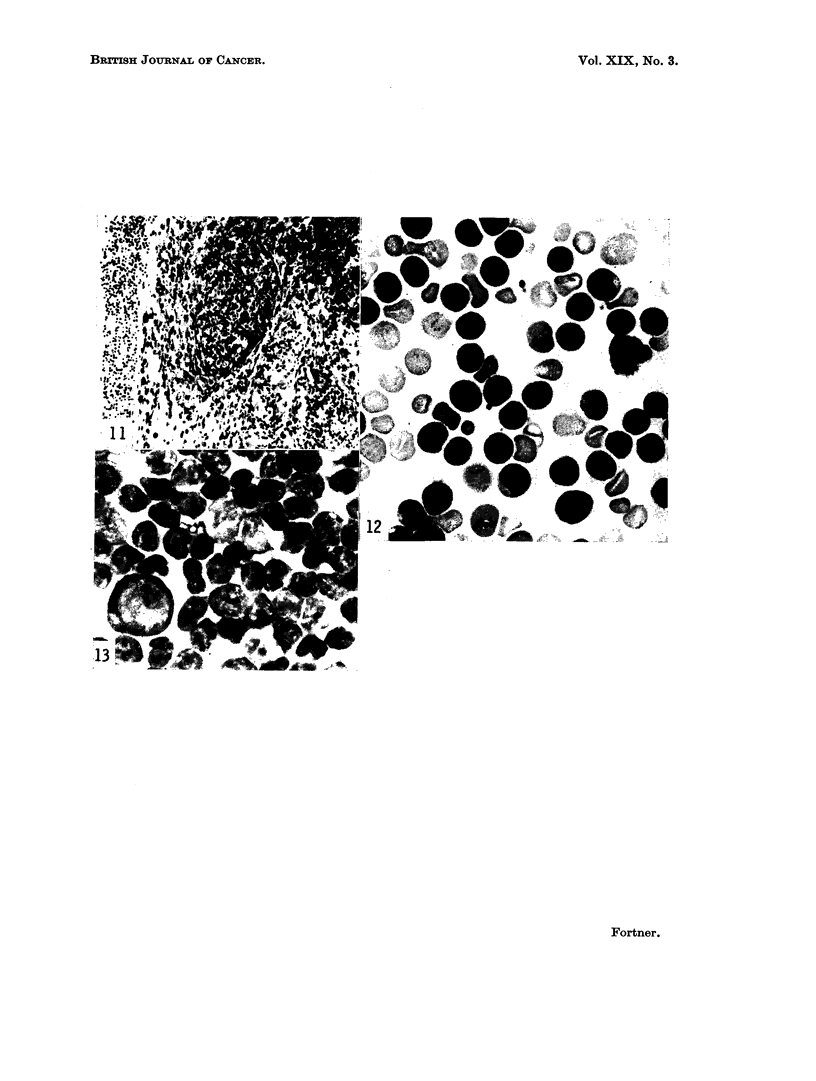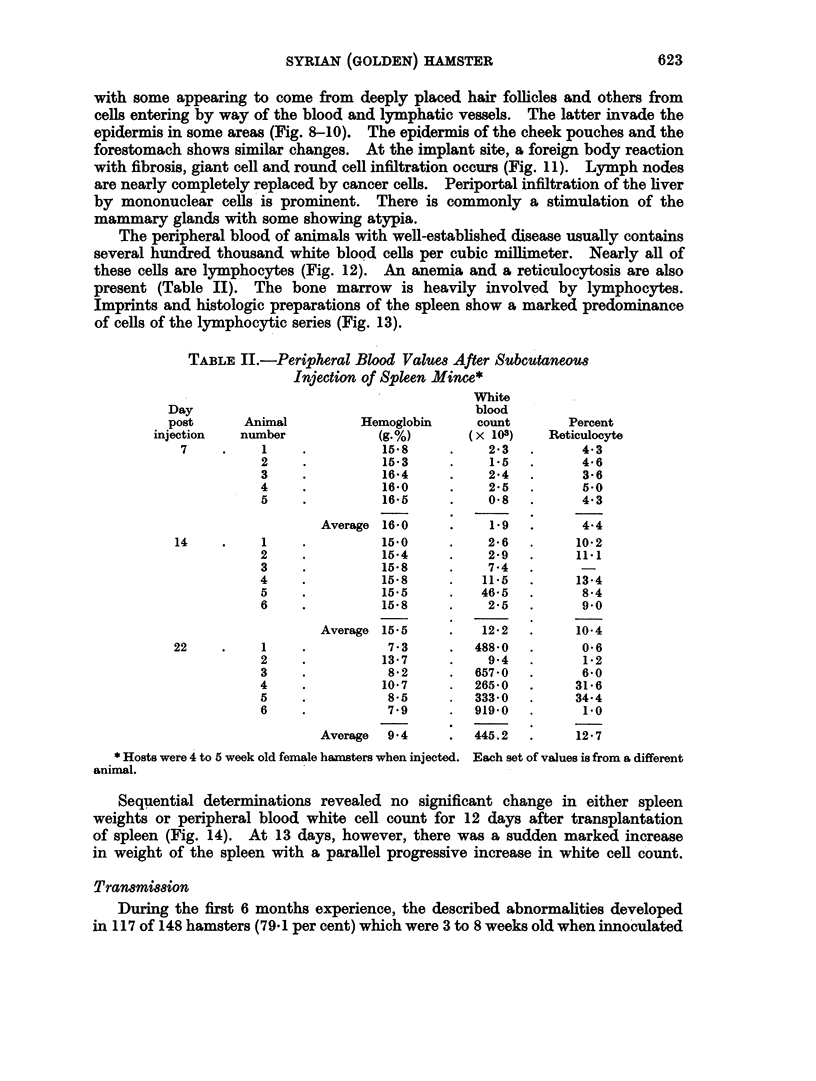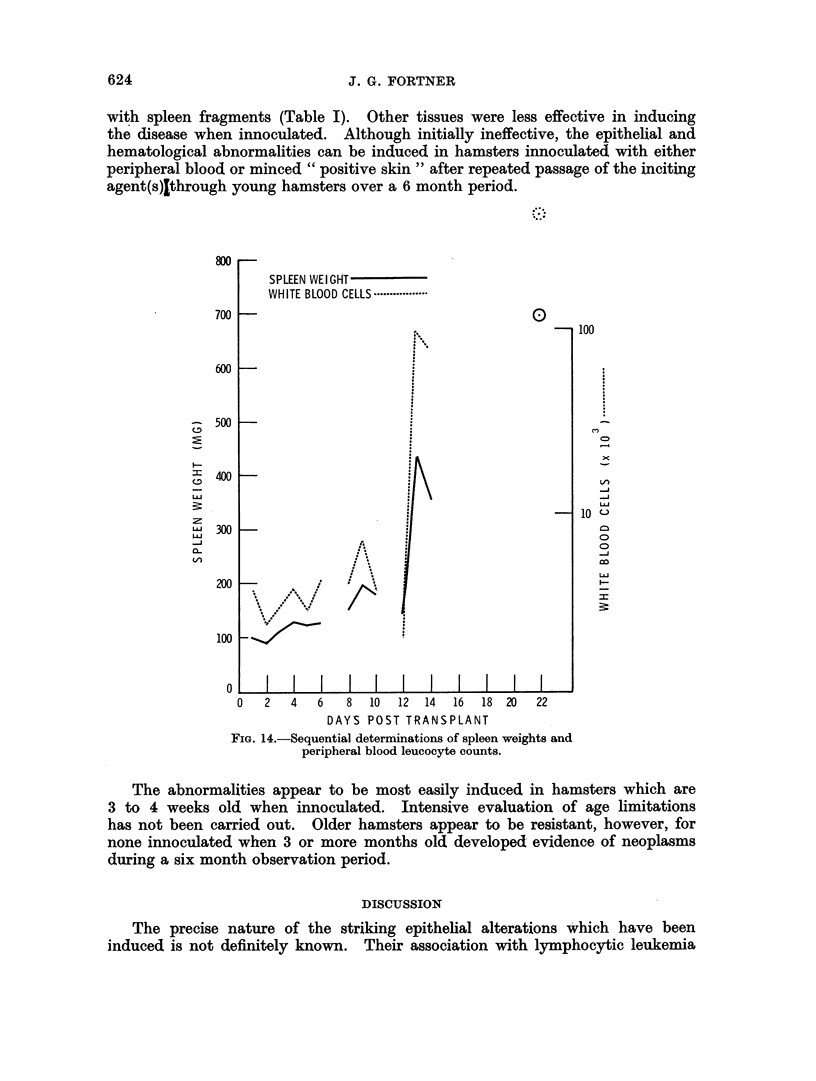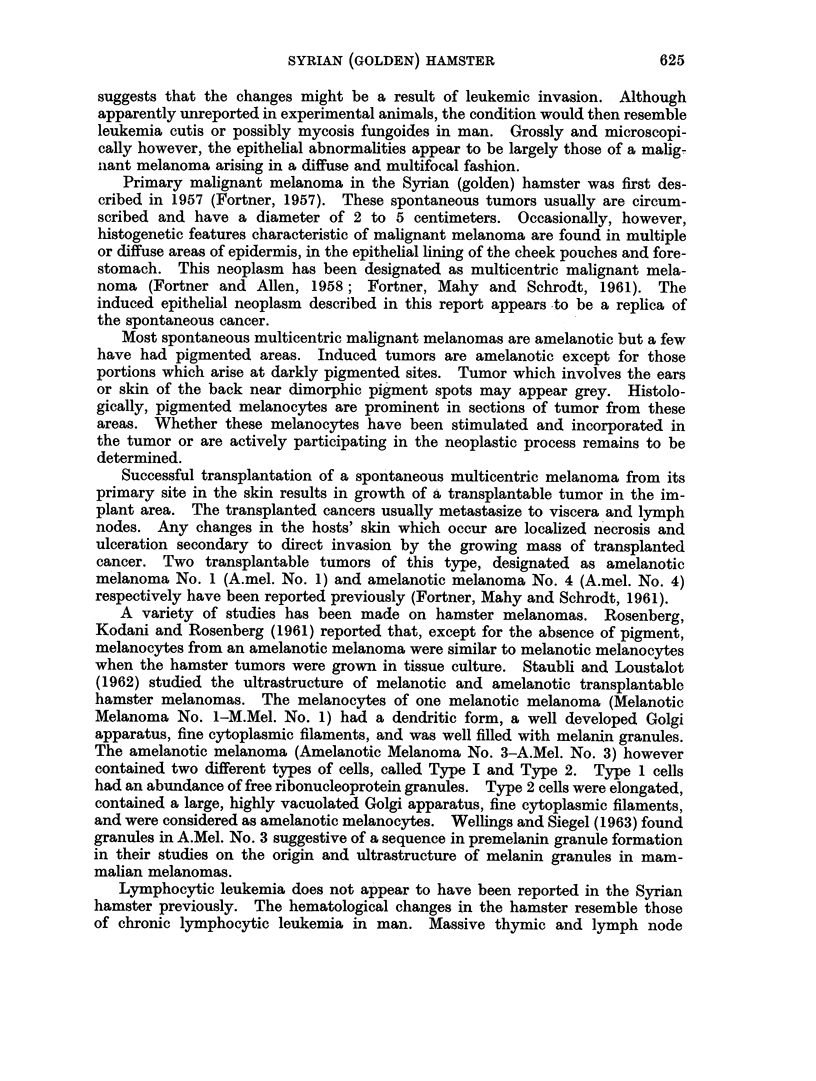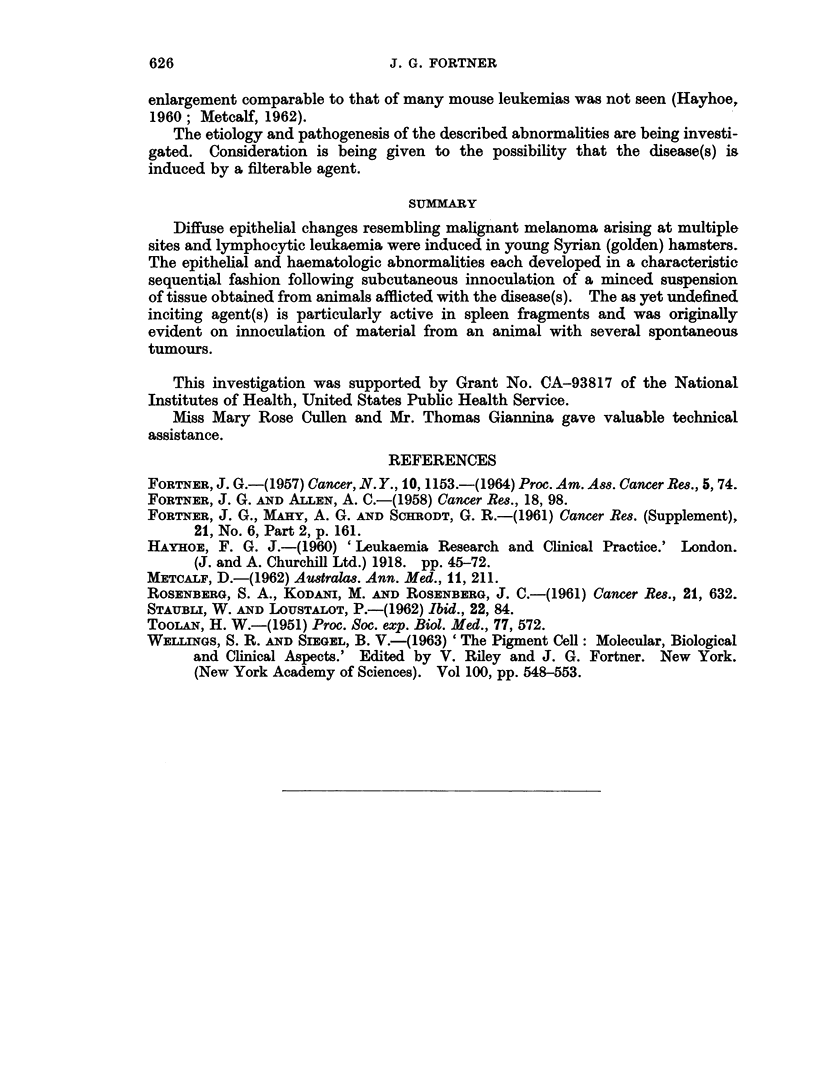# The production of skin lesions and lymphocytic leukemia in the Syrian (golden) hamster.

**DOI:** 10.1038/bjc.1965.78

**Published:** 1965-09

**Authors:** J. G. Fortner

## Abstract

**Images:**


### THE PRODUCTION OF SKIN LESIONS AND LYMPHOCYTIC LEUKEMIA IN THE SYRIAN (GOLDEN) HAMSTER

J. G. FORTNER*

*From the Division of Experimental Surgery and Physiology, Sloan-Kettering Institute and the Department of Surgery, Memorial Hospital, New York 21, New York*

Received for publication March 4, 1965

MALIGNANT epithelial and hematological abnormalities have been induced in young Syrian (golden) hamsters. The epithelial changes are diffuse and resemble those of malignant melanoma arising at multiple sites. Animals with well established epithelial changes usually have, in addition, a lymphocytic leukemia. The inciting agent(s) was originally obtained from the spleen of a hamster with spontaneous tumors. This report gives a general description of the phenomenon which has been referred to previously in abstract form (Fortner, 1964).

### MATERIALS AND METHODS

The initial source of active material was a male Syrian (golden) hamster which was killed when 28·5 months old. The animal had spontaneously developed a malignant melanoma which appeared to arise in a diffuse and multicentric fashion from the skin, cheek pouches and forestomach. (Fig. 1–6). The spleen weighed 890 mg. and was filled with mononuclear cells which seemed to be of the lymphocytic series on histological study. An adenomatous polyp in the gallbladder showed changes interpreted as *in situ* carcinoma. A blood count was not done but the marked lymphocytosis in a peripheral blood smear was compatible with a diagnosis of lymphocytic leukemia.

A mince suspension of the spleen was transplanted subcutaneously into two weanling male hamster. Fifty-four days later, both hosts showed pathological signs similar to the original donor animal except for the absence of a gallbladder tumor. Subsequently, the disease(s) has been serially propagated by implanting a suspension of minced spleen from afflicted animals into the subcutaneous tissue of 3 week old hamsters. The mince suspension technique as previously described (Toolan, 1951) has been used. Other viscera including liver, kidneys, brain, lymph nodes as well as skin involved by cancer and peripheral blood were transplanted using the same technique as for the spleen (Table I).

Hemogram values for animals in various stages of the disease were determined. Blood for analysis was drawn from the abdominal aorta after the animal was anesthetized with nembutal, administered intraperitoneally.

The relationship of spleen weight to the white blood cell count was determined using 70 female hamsters. The animals were 4 or 5 weeks old when innoculated with an active spleen mince. Five or 6 of the animals were killed at 1 or 2 day

* Recipient of Alfred P. Sloan Foundation Award in Cancer Research, 1964.

TABLE I.—*Comparative Effectiveness of Various Tissues in the Induction of Neoplasia*

| Number of animals | Number positive (%) | Tissue innoculated |
|---|---|---|
| 148 | 117 (79·1%) | spleen |
| 167 | 87 (52·0%) | liver |
| 24 | 6 (25·0%) | combined mince of kidney, brain, lung |
| 16 | 0 | skin* |
| 9 | 0 | thymus |
| 34 | 16 (47·0%) | lymph nodes |
| 8 | 0 | peripheral blood* |

* See text.

intervals thereafter for 14 days and again at 21 days. Each animal's leucocyte count and its spleen weight were determined. Average values were calculated and plotted in a semi-logarithmic manner.

Histologic studies have been made on formalin fixed tissue stained with hematoxylin and eosin. Fresh tissue imprints were stained with Giemsa.

Hamsters were obtained from the Lakeview Hamster Colony, Newfield, New Jersey. They were maintained under ordinary laboratory conditions. The animals were fed Purina Laboratory Chow and given a slice of fresh carrot daily. Tap water was available *ad libitum.*

### RESULTS

### *Onset and development*

In 3-week old hamsters, a characteristic and constant series of events develops when active minced spleen is transplanted subcutaneously. Seven to 10 days after innoculation, the animal's fur seems more erect than normal on the dorsolateral regions. Ventrally, the fur is roughened. There is a rosy hue to the animal's nose. Sneezing and rubbing of the nose occur frequently. There is slight prominence of the rims of the eyelids. The animals are hyperactive. They frequently pause to scratch and bite themselves as though afflicted with lice. Fine scaling and erythema of the skin is present. The skin is soft but slightly thickened at this time.

Most of the abnormalities are progressive so that 18 to 21 days after spleen injection, the animal's skin is wrinkled into transverse rolls, somewhat similar in contour to that of a washboard. The ears are quite black, scaly and have rounded edges. The markedly thickened skin appears too large for the animal. Scaling and hair loss is now severe. The animal moves about ponderously with a characteristic ventral flexion of the spine. If the animal survives, it becomes hairless. For a time, the animals seem to eat more than usual. Normal growth of young animals may appear retarded. In some instances, neoplastic disease is first manifested by changes on the lower legs, most pronounced on the inner aspect. Here, concentric rings of altered skin may progress to ulceration. Young animals developing an especially severe form of the disease may have wet necrosis of ventral skin with death rapidly ensuing. Ulceration and contraction of the skin may occur if animals survive long enough (Fig. 7). Most animals die within 30 days of onset of symptoms. Only an occasional animal lives more than 60 days.

*Morphologic features*

Necropsy of animals with well established disease reveals the skin to form a gray-tan rind 1–3 mm. thick which encases the animal's entire body. The skin is loosely attached to underlying tissues, there being no invasion of fascia or of skeletal muscle. Characteristically, the under surface of the skin is mottled bright red. The implant site of donor spleen is frequently not identifiable. Occasionally, it appears as a 2–5 mm. yellow-red nodule. Lymph nodes are 0·5 cm. to 1 cm. in diameter. The thymus is enlarged by tumor in about 50 per cent of animals. The liver is increased in size, coarsely granular and dark red to pale yellow. A splenomegaly is present with rare exception. In initial passages, however, spleen weights of hosts with well established skin changes did not exceed 150 mg. in over 20 per cent of animals. Splenomegaly is of two main types: (1) the spleen has a sharp edge and weighs 400 to 700 milligrams; (2) the spleen is huge weighing 1·5 to 2 or more grams and has a rounded edge. Spleens of both general types are dark red, friable and have a currant jelly consistency. The forestomach may be hemorrhagic and thickened.

Histologic study of the epidermis reveals multiple focal as well as diffuse areas in which the epidermal cells show loss of cohesion and other features of anaplasia which are reminiscent of primary malignant melanoma (Fortner and Allen, 1958). Various developmental stages from pre-invasive junctional changes through intraepithelial melanoma to an invasive neoplasm appear to be present. The findings are prominent at the periphery of hair follicles, as well as in the surface epithelium. Marked hyperkeratosis and dermal sclerosis are prominent. In the dermis, there is a diffuse infiltration of mononuclear cells having a clear vesicular nucleus and a prominent nucleolus. These seem to be of mixed origin

---

EXPLANATION OF PLATES

*Fig.* 1–6 : *Spontaneous multicentric melanoma.*

FIG. 1.—Ventral view of male hamster aged 22·2 months with spontaneous, primary multicentric melanoma. Changes in skin have progressed to ulceration in multiple focal areas.

FIG. 2.—Dorsal view of male hamster aged 22·9 months. Most of skin on back shows severe scaling and there are areas of ulceration.

FIG. 3.—Arrows point to prominent foci of multicentric melanoma in a female hamster aged 20·8 months. All of skin is thickened and there is fine scaling and wrinkling. Microscopically, malignant changes in the skin are diffuse.

FIG. 4.—Histological appearance of primary, multicentric melanoma. Note origin of cancer cells from basilar layer of epidermis. Changes in hair follicle (left of centre in illustration) may explain hair loss of animals with the disease. Pigmented melanocytes evident in lower right of photograph. ×200.

FIG. 5.—Spreading of cells from hair follicle into dermis. Dermal sclerosis is marked. ×180.

FIG. 6.—Junctional changes and hyperkeratosis are particularly evident. ×175.

*Fig.* 7–13 : *Induced abnormalities.*

FIG. 7.—Advanced changes in the skin.

FIG. 8.—Histological appearance of an area of skin where mononuclear cells infiltrate the epidermis as well as upper layer of the dermis. ×12.

FIG. 9 and 10.—Cells in other areas of the epidermis show loss of cohesion and anaplasia compatible with the diagnosis of primary malignant melanoma. ×200.

FIG. 11.—Cellular reaction to spleen fragments implanted subcutaneously. Foreign body giant cells, fibrosis and round cell infiltration are evident. ×90.

FIG. 12.—Peripheral blood smear of animal with induced epithelial abnormalities and a lymphocytic leukemia. ×700.

FIG. 13.—Imprint of spleen of animal with the induced neoplasms. ×700

Fortner.

Fortner.

Fortner.

Fortner.

with some appearing to come from deeply placed hair follicles and others from cells entering by way of the blood and lymphatic vessels. The latter invade the epidermis in some areas (Fig. 8–10). The epidermis of the cheek pouches and the forestomach shows similar changes. At the implant site, a foreign body reaction with fibrosis, giant cell and round cell infiltration occurs (Fig. 11). Lymph nodes are nearly completely replaced by cancer cells. Periportal infiltration of the liver by mononuclear cells is prominent. There is commonly a stimulation of the mammary glands with some showing atypia.

The peripheral blood of animals with well-established disease usually contains several hundred thousand white blood cells per cubic millimeter. Nearly all of these cells are lymphocytes (Fig. 12). An anemia and a reticulocytosis are also present (Table II). The bone marrow is heavily involved by lymphocytes. Imprints and histologic preparations of the spleen show a marked predominance of cells of the lymphocytic series (Fig. 13).

TABLE II.—*Peripheral Blood Values After Subcutaneous Injection of Spleen Mince**

| Day post injection | Animal number | | Hemoglobin (g.%) | White blood count (× $10^3$) | Percent Reticulocyte |
|---|---|---|---|---|---|
| 7 | 1 | | 15·8 | 2·3 | 4·3 |
| | 2 | | 15·3 | 1·5 | 4·6 |
| | 3 | | 16·4 | 2·4 | 3·6 |
| | 4 | | 16·0 | 2·5 | 5·0 |
| | 5 | | 16·5 | 0·8 | 4·3 |
| | | Average | 16·0 | 1·9 | 4·4 |
| 14 | 1 | | 15·0 | 2·6 | 10·2 |
| | 2 | | 15·4 | 2·9 | 11·1 |
| | 3 | | 15·8 | 7·4 | — |
| | 4 | | 15·8 | 11·5 | 13·4 |
| | 5 | | 15·5 | 46·5 | 8·4 |
| | 6 | | 15·8 | 2·5 | 9·0 |
| | | Average | 15·5 | 12·2 | 10·4 |
| 22 | 1 | | 7·3 | 488·0 | 0·6 |
| | 2 | | 13·7 | 9·4 | 1·2 |
| | 3 | | 8·2 | 657·0 | 6·0 |
| | 4 | | 10·7 | 265·0 | 31·6 |
| | 5 | | 8·5 | 333·0 | 34·4 |
| | 6 | | 7·9 | 919·0 | 1·0 |
| | | Average | 9·4 | 445.2 | 12·7 |

* Hosts were 4 to 5 week old female hamsters when injected. Each set of values is from a different animal.

Sequential determinations revealed no significant change in either spleen weights or peripheral blood white cell count for 12 days after transplantation of spleen (Fig. 14). At 13 days, however, there was a sudden marked increase in weight of the spleen with a parallel progressive increase in white cell count.

*Transmission*

During the first 6 months experience, the described abnormalities developed in 117 of 148 hamsters (79·1 per cent) which were 3 to 8 weeks old when innoculated with spleen fragments (Table I). Other tissues were less effective in inducing the disease when innoculated. Although initially ineffective, the epithelial and hematological abnormalities can be induced in hamsters innoculated with either peripheral blood or minced " positive skin " after repeated passage of the inciting agent(s) through young hamsters over a 6 month period.

FIG. 14.—Sequential determinations of spleen weights and peripheral blood leucocyte counts.

The abnormalities appear to be most easily induced in hamsters which are 3 to 4 weeks old when innoculated. Intensive evaluation of age limitations has not been carried out. Older hamsters appear to be resistant, however, for none innoculated when 3 or more months old developed evidence of neoplasms during a six month observation period.

### DISCUSSION

The precise nature of the striking epithelial alterations which have been induced is not definitely known. Their association with lymphocytic leukemia suggests that the changes might be a result of leukemic invasion. Although apparently unreported in experimental animals, the condition would then resemble leukemia cutis or possibly mycosis fungoides in man. Grossly and microscopically however, the epithelial abnormalities appear to be largely those of a malignant melanoma arising in a diffuse and multifocal fashion.

Primary malignant melanoma in the Syrian (golden) hamster was first described in 1957 (Fortner, 1957). These spontaneous tumors usually are circumscribed and have a diameter of 2 to 5 centimeters. Occasionally, however, histogenetic features characteristic of malignant melanoma are found in multiple or diffuse areas of epidermis, in the epithelial lining of the cheek pouches and forestomach. This neoplasm has been designated as multicentric malignant melanoma (Fortner and Allen, 1958; Fortner, Mahy and Schrodt, 1961). The induced epithelial neoplasm described in this report appears to be a replica of the spontaneous cancer.

Most spontaneous multicentric malignant melanomas are amelanotic but a few have had pigmented areas. Induced tumors are amelanotic except for those portions which arise at darkly pigmented sites. Tumor which involves the ears or skin of the back near dimorphic pigment spots may appear grey. Histologically, pigmented melanocytes are prominent in sections of tumor from these areas. Whether these melanocytes have been stimulated and incorporated in the tumor or are actively participating in the neoplastic process remains to be determined.

Successful transplantation of a spontaneous multicentric melanoma from its primary site in the skin results in growth of a transplantable tumor in the implant area. The transplanted cancers usually metastasize to viscera and lymph nodes. Any changes in the hosts' skin which occur are localized necrosis and ulceration secondary to direct invasion by the growing mass of transplanted cancer. Two transplantable tumors of this type, designated as amelanotic melanoma No. 1 (A.mel. No. 1) and amelanotic melanoma No. 4 (A.mel. No. 4) respectively have been reported previously (Fortner, Mahy and Schrodt, 1961).

A variety of studies has been made on hamster melanomas. Rosenberg, Kodani and Rosenberg (1961) reported that, except for the absence of pigment, melanocytes from an amelanotic melanoma were similar to melanotic melanocytes when the hamster tumors were grown in tissue culture. Staubli and Loustalot (1962) studied the ultrastructure of melanotic and amelanotic transplantable hamster melanomas. The melanocytes of one melanotic melanoma (Melanotic Melanoma No. 1–M.Mel. No. 1) had a dendritic form, a well developed Golgi apparatus, fine cytoplasmic filaments, and was well filled with melanin granules. The amelanotic melanoma (Amelanotic Melanoma No. 3–A.Mel. No. 3) however contained two different types of cells, called Type I and Type 2. Type 1 cells had an abundance of free ribonucleoprotein granules. Type 2 cells were elongated, contained a large, highly vacuolated Golgi apparatus, fine cytoplasmic filaments, and were considered as amelanotic melanocytes. Wellings and Siegel (1963) found granules in A.Mel. No. 3 suggestive of a sequence in premelanin granule formation in their studies on the origin and ultrastructure of melanin granules in mammalian melanomas.

Lymphocytic leukemia does not appear to have been reported in the Syrian hamster previously. The hematological changes in the hamster resemble those of chronic lymphocytic leukemia in man. Massive thymic and lymph node enlargement comparable to that of many mouse leukemias was not seen (Hayhoe, 1960; Metcalf, 1962).

The etiology and pathogenesis of the described abnormalities are being investigated. Consideration is being given to the possibility that the disease(s) is induced by a filterable agent.

### SUMMARY

Diffuse epithelial changes resembling malignant melanoma arising at multiple sites and lymphocytic leukaemia were induced in young Syrian (golden) hamsters. The epithelial and haematologic abnormalities each developed in a characteristic sequential fashion following subcutaneous innoculation of a minced suspension of tissue obtained from animals afflicted with the disease(s). The as yet undefined inciting agent(s) is particularly active in spleen fragments and was originally evident on innoculation of material from an animal with several spontaneous tumours.

This investigation was supported by Grant No. CA–93817 of the National Institutes of Health, United States Public Health Service.

Miss Mary Rose Cullen and Mr. Thomas Giannina gave valuable technical assistance.

### REFERENCES

Fortner, J. G.—(1957) *Cancer, N.Y.*, **10**, 1153.—(1964) *Proc. Am. Ass. Cancer Res.*, **5**, 74.
Fortner, J. G. and Allen, A. C.—(1958) *Cancer Res.*, 18, 98.
Fortner, J. G., Mahy, A. G. and Schrodt, G. R.—(1961) *Cancer Res.* (Supplement), **21**, No. 6, Part 2, p. 161.
Hayhoe, F. G. J.—(1960) 'Leukaemia Research and Clinical Practice.' London. (J. and A. Churchill Ltd.) 1918. pp. 45–72.
Metcalf, D.—(1962) *Australas. Ann. Med.*, **11**, 211.
Rosenberg, S. A., Kodani, M. and Rosenberg, J. C.—(1961) *Cancer Res.*, **21**, 632.
Staubli, W. and Loustalot, P.—(1962) *Ibid.*, **22**, 84.
Toolan, H. W.—(1951) *Proc. Soc. exp. Biol. Med.*, **77**, 572.
Wellings, S. R. and Siegel, B. V.—(1963) 'The Pigment Cell: Molecular, Biological and Clinical Aspects.' Edited by V. Riley and J. G. Fortner. New York. (New York Academy of Sciences). Vol 100, pp. 548–553.